# Natural history of *Mycobacterium fortuitum* pulmonary infection presenting with migratory infiltrates: a case report with microbiological analysis

**DOI:** 10.1186/s12879-017-2892-9

**Published:** 2018-01-02

**Authors:** Satoshi Okamori, Takanori Asakura, Tomoyasu Nishimura, Eiko Tamizu, Makoto Ishii, Mitsunori Yoshida, Hanako Fukano, Yuichiro Hayashi, Masaki Fujita, Yoshihiko Hoshino, Tomoko Betsuyaku, Naoki Hasegawa

**Affiliations:** 1 Division of Pulmonary Medicine, Department of Medicine, Keio University School of Medicine, 35 Shinanomachi, Shinjuku, Tokyo, 160-8582 Japan; 2 Keio University Health Center, 35 Shinanomachi, Shinjuku, Tokyo, 160-8582 Japan; 3 Department of Mycobacteriology, Leprosy Research Center, National Institute of Infectious Diseases, 4-2-1 Aobacho, Higashimurayama, Tokyo, 189-0002 Japan; 4 Division of Diagnostic Pathology, Keio University School of Medicine, 35 Shinanomachi, Shinjuku, Tokyo, 160-8582 Japan; 5 Department of Respiratory Medicine, Faculty of Medicine, Fukuoka University, 7-45-1 Nanakuma, Jonan-ku, Fukuoka, 814-0180 Japan; 6 Center for Infectious Diseases and Infection Control, Keio University School of Medicine, 35 Shinanomachi, Shinjuku, Tokyo, 160-8582 Japan

**Keywords:** Nontuberculous mycobacteria (NTM), Rapidly growing mycobacteria (RGM), Aspiration, Mycobacterial infection, Lipoid pneumonia

## Abstract

**Background:**

Presence of *Mycobacterium fortuitum* in respiratory tracts usually indicates mere colonization or transient infection, whereas true pulmonary infection occurs in patients with gastroesophageal disease. However, little is known about the diagnostic indications for true *M. fortuitum* pulmonary infection and the natural history of the disease.

**Case presentation:**

A 59-year-old man was referred to our hospital for treatment against *M. fortuitum* pulmonary infection. Fifteen years before the referral, he underwent total gastrectomy, after which he experienced esophageal reflux symptoms. After the referral, the patient was closely monitored without antimicrobial therapy because of mild symptoms and no pathological evidence of *M. fortuitum* pulmonary infection. During the observation, chest imaging showed migratory infiltrates. Two years after the referral, his lung biopsy specimen revealed foamy macrophages and multinucleated giant cells, indicating lipoid pneumonia. However, he was continually monitored without any treatment because there was no evidence of nontuberculous mycobacterial infection. Four years after the referral, he developed refractory pneumonia despite receiving adequate antibiotic therapy. After confirmation of granulomatous lesions, multiple antimicrobial therapy for *M. fortuitum* resulted in a remarkable improvement with no exacerbation for over 5 years. Random amplified polymorphic DNA polymerase chain reaction analysis revealed identical *M. fortuitum* strains in seven isolates from six sputum and one intestinal fluid specimens obtained during the course of the disease.

**Conclusions:**

We have described a patient with *M. fortuitum* pulmonary infection who presented with migratory infiltrates. The pathological evidence and microbiological analysis suggested that *M. fortuitum* pulmonary infection was associated with lipoid pneumonia and chronic exposure to gastrointestinal fluid. Therefore, physicians should carefully monitor patients with *M. fortuitum* detected from lower respiratory tract specimens and consider antimicrobial therapy for *M. fortuitum* infection when the patient does not respond to adequate antibiotic therapy against common pneumonia pathogens.

## Background

Nontuberculous mycobacterial (NTM) infection, mainly causing pulmonary infections, has increasingly become a socially important disease affecting not only health-related quality of life but also the prognosis of patients [1, 2]. Recent epidemiological studies have revealed increased incidence of NTM infections worldwide, including in Japan [3, 4].

NTM are ubiquitous organisms, isolated from environmental sources such as water and soil [5]. Therefore, physicians sometimes have difficulty distinguishing true infection from contamination or colonization, especially one caused by rare species. *Mycobacterium fortuitum* is one of the rapidly growing mycobacteria, which are mainly present in soil and water [6, 7]. *M. fortuitum* mainly causes skin and bone/joint infections in both immunocompetent and immunocompromised patients [5].

Many patients with *M. fortuitum* detected from lower respiratory tract specimens were found to have underlying lung diseases, including old pulmonary tuberculosis, lung cancer, interstitial lung disease, and other NTM pulmonary diseases [8]. Most of these patients did not require long-term antimicrobial therapy despite continuous detection of *M. fortuitum*. Thus, *M. fortuitum* from respiratory tracts has been considered to indicate mere colonization or transient infection [8]. On the other hand, true pulmonary infection occurs in patients with gastroesophageal disease [5]. However, little is known about the diagnostic indications for true *M. fortuitum* pulmonary infection and the natural history of the disease. Therefore, the decision to administer multiple antimicrobial therapy remains a clinical issue for physicians.

We herein describe a postgastrectomy patient with *M. fortuitum* pulmonary infection complicated by chronic aspiration who presented with migratory infiltrates, developed refractory pneumonia with evidence of granulomatous lesions, and underwent subsequent multiple antimicrobial therapy. Furthermore, we will show microbiological evidence among specimens from the respiratory and upper digestive tracts.

## Case presentation

A 59-year-old man was referred to our hospital for treatment against *M. fortuitum* pulmonary infection. He underwent total gastrectomy for gastric cancer 15 years before the referral. After the surgery, he often had esophageal reflux symptoms and occasional episodes of vomiting. He was a current smoker of 25 pack-years and then quit smoking after referral. He did not use oily substances as constipation drugs or nose drops. Three years before the referral, he was diagnosed with *M. fortuitum* pulmonary infection according to sputum culture results, after which multiple antimicrobial therapy with rifampicin, ethambutol, clarithromycin, and levofloxacin was administered for a year. Eight months before the referral, treatment with isoniazid, rifampicin, ethambutol, and clarithromycin was reinstituted for the relapse; however, pulmonary involvement did not resolve.

**Fig. 1:**
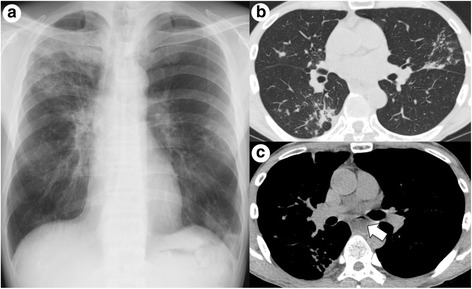
**a** Chest imaging performed during the referral showed infiltrates in the right upper and left lower lung fields and bilateral middle fields. **b** Computed tomography images showed consolidations and nodular shadows in multiple lung lobes. **c** The esophagus was dilated with food residue (arrow)

At the time of referral to our hospital, he only had an intermittent cough and sputum. Chest radiograph showed consolidations in the right upper and left lower lung fields and nodular shadows in the middle bilaterally (Fig. 1a). Chest computed tomography (CT) revealed consolidations in the right upper and left lower lobes, nodular shadows in the right middle and lower lobes and left lingua (Fig. 1b), and dilated esophagus with food residue (Fig. 1c). Laboratory data including lymphocyte count, gamma globulin level, and anti-HIV antibody level were unremarkable. Although the sputum culture was positive for mycobacteria species identified as *M. fortuitum*, cultures from the transbronchial lung biopsy (TBLB) specimen were negative. Histological examination only showed nonspecific inflammation such as exudation of fibrin without granulomatous lesions. Treatment with rabeprazole for his esophageal reflux symptoms was administered, and he was followed up without treatment with antibiotics.

**Fig. 2:**
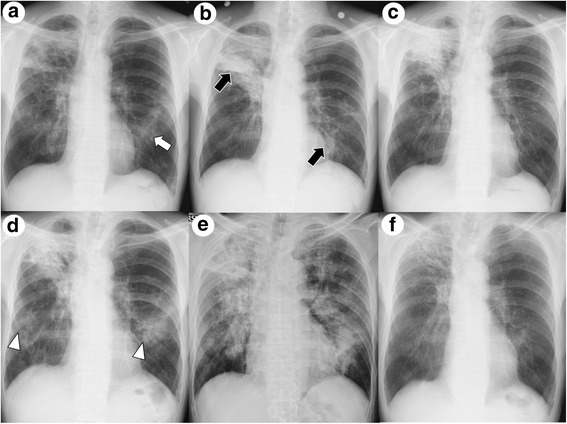
Chest imaging performed during the observation. **a** At 6 months after the referral. New infiltrates appeared in the left middle field (white arrow). **b** At 14 months after the referral. The consolidation in the right upper field was deteriorated, and the infiltrates in the left middle field migrated toward the lower right (black arrows). **c** At 25 months after the referral. The infiltrations in the left lung field were improved without treatment. **d** At 28 months after the referral. New infiltrates appeared bilaterally (white arrowhead) **e** At 38 months after the referral. The patients developed apparent aspiration pneumonia, with chest radiograph showing bilateral consolidations. **f** At 5 years after treatment against *M. fortuitum* was started. Multiple pulmonary lesions improved

**Fig. 3:**
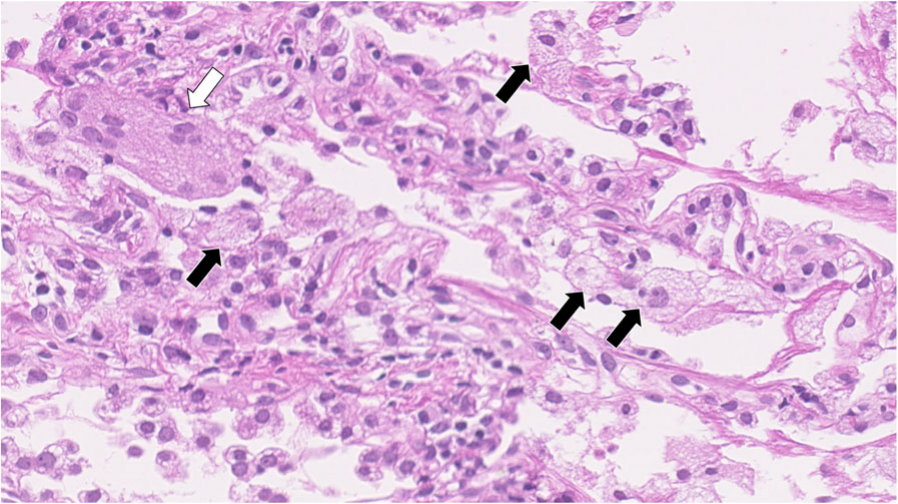
Photomicrographs of computed tomography-guided lung biopsy specimens showing foamy macrophages in the alveolar spaces (black arrows) and multinucleated giant cells (white arrow)

During the period following his referral, he occasionally had cough and sputum without fever and weight loss. Chest imaging showed migratory infiltrates (Fig. 2a). Although the sputum culture was continually positive for *M. fortuitum*, his symptoms did not worsen. At 14 months after his referral, we performed TBLB due to deterioration of consolidation in the right upper lung field (Fig. 2b). Cultures from the TBLB specimen were negative, and histological examination of the specimen revealed no remarkable findings for *M. fortuitum* infection. To investigate for evidence of NTM disease, CT-guided lung biopsy was also performed. The lung biopsy specimen revealed foamy macrophages and multinucleated giant cells, indicating lipoid pneumonia (Fig. 3). The patient was continually monitored without any treatment because there was no evidence of NTM infection.

**Fig. 4:**
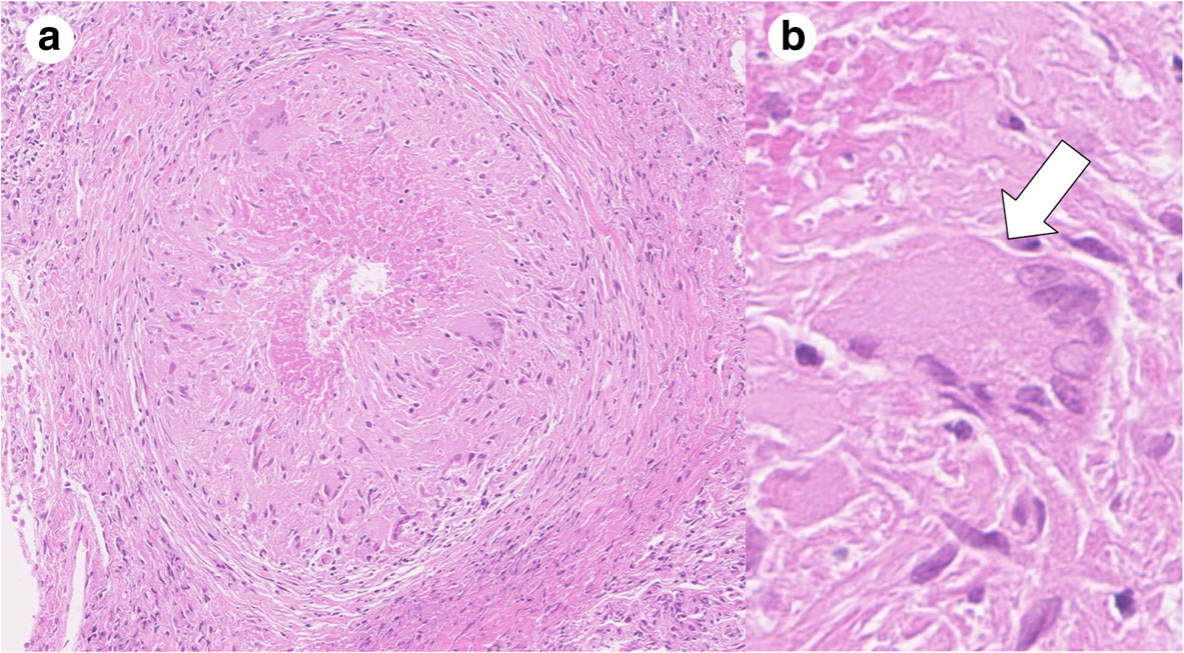
Photomicrographs of transbronchial lung biopsy specimens. **a** An epithelioid granuloma with necrosis. **b** Langhans giant cells (white arrow)

After the investigation, chest imaging continually showed migratory infiltrates (Fig. 2c, d). At 38 months after his referral, he developed aspiration pneumonia after an episode of vomiting (Fig. 2e). Even though his inflammatory response improved due to antibiotic treatment with meropenem followed by ampicillin sulbactum, the consolidations remained. Esophagogastroduodenoscopy revealed a large amount of food residue, which made it difficult to observe the esophageal mucosa. Treatment with camostat and mosapride improved his reflux symptoms partially, but the migratory infiltrates did not resolve. TBLB was performed again, and histological examination of the specimen revealed granulomatous lesions with necrosis (Fig. 4), indicating NTM infection. Meanwhile, *M. fortuitum* was detected through microbiological analysis of cultures from sputum and intestinal fluid. Multiple antimicrobial therapy with imipenem/cilastatin (for 2 weeks), amikacin (for 3 months), clarithromycin, minocycline, and levofloxacin was then administered. The treatment improved his cough and sputum, as well as his pulmonary lesions. He continued the treatment for 5 years, during which the migratory infiltrates did not recur (Fig. 2f). The clinical course of the treatment is shown in Table 1.

**Table 1:** Clinical course of the treatment

Treatment regimen	Start time	Duration	Note
RIF + EB + CLA + LVFX	3 years before the referral	1 year	
RIF + EB + CLA + INH	8 months before the referral	8 months	
MEPM → ABPC/SBT	38 months after the referral	2 weeks	Treatment for aspiration pneumonia
IPM/CS (for 2 weeks) + AMK (for 3 months) + CLA + MINO + LVFX	40 months after the referral	5 years (continuing)	Improvement and no recurrence of migratory infiltrates

Abbreviations: *RIF* rifampicin, *EB* ethambutol, *CLA* clarithromycin, *LVFX* levofloxacin; *INH* isoniazid, *MEPM* meropenem, *ABPC/SBT* ampicillin/sulbactum, *IPM/CS*, imipenem/cilastatin, *AMK* amikacin, *MINO*, minomycin

**Fig. 5:**
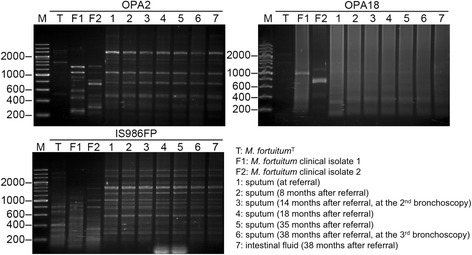
Randomly amplified polymorphic DNA polymerase chain reaction (RAPD-PCR) analysis with mycobacteria-specific primers as described in a previous study [23, 24]. Lane M, 100-bp DNA ladder size markers; lane T, type strain of *M. fortuitum* (ATCC 6841 strain); lanes F1 and F2, *M. fortuitum* clinical isolates from the cultures of other patients; lanes 1–7, *M. fortuitum* isolated from our patient (1–6, sputum; 7, intestinal fluid) RAPD-PCR patterns produced with primers OPA2, OPA18, and IS986FP are shown. Strains were determined to be identical when the same band patterns were observed with the three primers or one major band difference was observed in only one of the three primers

The microbiological analysis was performed using random amplified polymorphic DNA polymerase chain reaction (RAPD-PCR) analysis, which revealed identical *M. fortuitum* strains in seven isolates from six sputum and one intestinal fluid specimens during the course of the disease (Fig. 5). The drug sensitivity of these strains is shown in Table 2. There was no evidence of development of resistance during the course of the case.

**Table 2:** Drug sensitivity of the isolated strains

Strain	Antibiotics												Isolated from
	CLA	RIF	STFX	LVFX	EB	AMK	FRPM	DRPM	INH	MINO	IPM	TGC	
*M. fortuitum* type strain	4	>32	1	≦0.25	0.5	2	64	128	4	≦1	>64	0.5	sputum
*M. fortuitum* #1	64	>32	0.25	≦0.25	0.5	8	2	32	4	4	>64	0.5	sputum (at referral)
*M. fortuitum* #2	64	>32	0.125	≦0.25	0.5	4	4	32	8	16	>64	0.5	sputum (8 months after referral)
*M. fortuitum* #3	32	>32	0.5	≦0.25	0.5	8	2	32	8	16	>64	0.5	sputum (14 months after referral, at the 2nd bronchoscopy)
*M. fortuitum* #4	128	>32	0.25	≦0.25	0.5	4	2	32	4	8	>64	0.5	sputum (18 months after referral)
*M. fortuitum* #5	32	>32	0.125	≦0.25	0.5	4	4	32	8	16	>64	0.5	sputum (35 months after referral)
*M. fortuitum* #6	32	>32	0.125	≦0.25	0.5	4	4	32	8	16	>64	0.5	sputum (38 months after referral, at the 3rd bronchoscopy)
*M. fortuitum* #7	32	>32	0.125	≦0.25	0.5	4	4	64	8	8	>64	0.5	intestinal fluid (38 months after referral)

The isolates were incubated with cation-adjusted Mueller-Hinton broth at 30 °C. Abbreviations: *CLA* clarithromycin, *RIF* rifampicin, *STFX* sitafloxacin, *LVFX* levofloxacin, *EB* ethambutol, *AMK* amikacin, *FRPM* faropenem, *DRPM* doripenem, *INH*, isoniazid, *MINO* minomycin, *IPM* imipenem, *TGC* tigecycline

## Discussion and conclusions

Previous studies revealed that *M. fortuitum* causes pulmonary infection requiring antimicrobial therapy in patients with gastroesophageal diseases, especially esophagus achalasia [9]. Furthermore, patients with other NTM pulmonary diseases have a high prevalence of increased esophageal acid exposure [10]. Although the pathogenesis of *M. fortuitum* pulmonary infection in patients is unclear, we have confirmed its association with lipoid pneumonia and the evidence of identical *M. fortuitum* strains among all the sputum and intestinal fluid specimens in our patient.

Lipoid pneumonia is caused by aspirating fatlike compounds of animal, vegetable, or mineral origin [11]. Previous studies demonstrated that *M. fortuitum* have an advantage for survival in a lipid environment [12–14]. Notably, trehalose-6,6′-dimycolate (TDM), one of the many known mycolates contained in virulent strains of mycobacteria including *Mycobacterium tuberculosis* and *M. fortuitum* [15], has important roles with lipids. In mouse experiments, TDM-induced granuloma was only induced when TDM was inoculated in a lipid environment [12]. Therefore, in our case, pathologically confirmed lipoid pneumonia may be associated with the pathogenesis of *M. fortuitum* pulmonary infection.

To our knowledge, we are the first to report on identical *M. fortuitum* strains among cultures from sputum and intestinal fluid specimens using RAPD-PCR analysis. Furthermore, we have revealed identical strains among all the sputum specimens in the course of the disease. The thoracic digestive tract, which is highly nourished and has optimal temperature, is a suitable environment for *M. fortuitum* growth, and it may be suggested that a considerable amount of bacteria are present in the tract’s contents [16]. Although the exact pathogenesis is difficult to prove, we speculate that chronic exposure to gastrointestinal fluid may have caused the pathogenesis of *M. fortuitum* pulmonary infection, even though positive gastrointestinal fluid culture might simply be the result of swallowing respiratory secretions containing *M. fortuitum*.

We are also the first to describe the natural history of *M. fortuitum* pulmonary infection caused by identical strains and showing migratory infiltrates. In a previous study, the CT findings of *M. fortuitum* pulmonary infection demonstrated various lesions, including bronchiectasis, nodules, consolidation, and cavity lesions [8]; however, the longitudinal changes without antimicrobial therapy have not been reported. Various pulmonary diseases such as cryptogenic organizing pneumonia, chronic eosinophilic pneumonia, hypersensitivity pneumonitis, allergic bronchopulmonary aspergillosis, lymphoproliferative disorder, vasculitis, and aspiration pneumonitis can cause migratory infiltrates [17–20]. Furthermore, gastroesophageal reflux can introduce pulmonary infiltrates, leading to aspiration pneumonitis [21]. In view of NTM infection, one case on *Mycobacterium abscessus* complex pulmonary infection has also shown migratory infiltrates [22]. Moreover, *M. fortuitum* is a pathogen that can survive in the lipid environment caused by aspiration as previously mentioned. In our case, treatment for esophageal reflux with rabeprazole, camostat, and mosapride did not improve his migratory infiltrates. Moreover, multiple antimicrobial therapy for *M. fortuitum* resulted in improvement with no relapse. Based on these findings, we consider that migratory infiltrates were probably caused by *M. fortuitum* infection related to aspiration due to esophageal reflux. Although the choice of suitable antimicrobial therapy was unclear, the patient achieved improvement, even after the confirmation of granulomatous lesions with refractory pneumonia despite adequate antibiotic therapy.

In conclusion, we have described a patient with *M. fortuitum* pulmonary infection who presented with migratory infiltrates, and the pathological evidence and microbiological analysis suggested that *M. fortuitum* pulmonary infection was associated with lipoid pneumonia and chronic exposure to gastrointestinal fluid. Hence, physicians should carefully monitor patients with *M. fortuitum* detected from lower respiratory tract specimens, and consider antimicrobial therapy for *M. fortuitum* when the patient does not respond to adequate antibiotic therapy against common pneumonia pathogens.

## Abbreviations

CT: Computed tomography
NTM: Nontuberculous mycobacteria
RAPD-PCR: Random amplified polymorphic DNA polymerase chain reaction
TBLB: Transbronchial lung biopsy
TDM: Trehalose-6,6′-dimycolate

## References

[1] Asakura T, Funatsu Y, Ishii M, Namkoong H, Yagi K, Suzuki S (2015). Health-related quality of life is inversely correlated with C-reactive protein and age in Mycobacterium Avium Complex lung disease: a cross-sectional analysis of 235 patients. Respir Res.

[2] Morimoto K, Iwai K, Uchimura K, Okumura M, Yoshiyama T, Yoshimori K, Ogata H (2014). A steady increase in nontuberculous mycobacteriosis mortality and estimated prevalence in Japan. Ann Am Thorac Soc.

[3] Namkoong H, Kurashima A, Morimoto K, Hoshino Y, Hasegawa N, Ato M (2016). Epidemiology of pulmonary Nontuberculous Mycobacterial disease, Japan(1). Emerg Infect Dis.

[4] Prevots DR, Marras TK (2015). Epidemiology of human pulmonary infection with nontuberculous mycobacteria: a review. Clin Chest Med.

[5] Griffith DE, Aksamit T, Brown-Elliott BA, Catanzaro A, Daley C, Gordin F (2007). An official ATS/IDSA statement: diagnosis, treatment, and prevention of nontuberculous mycobacterial diseases. Am J Respir Crit Care Med.

[6] Goslee S, Wolinsky E (1976). Water as a source of potentially pathogenic mycobacteria. Am Rev Respir Dis.

[7] Wolinsky E, Rynearson TK (1968). Mycobacteria in soil and their relation to disease-associated strains. Am Rev Respir Dis.

[8] Park S, Suh GY, Chung MP, Kim H, Kwon OJ, Lee KS (2008). Clinical significance of mycobacterium fortuitum isolated from respiratory specimens. Respir Med.

[9] Hadjiliadis D, Adlakha A, Prakash UB (1999). Rapidly growing mycobacterial lung infection in association with esophageal disorders. Mayo Clin Proc.

[10] Koh WJ, Lee JH, Kwon YS, Lee KS, Suh GY, Chung MP (2007). Prevalence of gastroesophageal reflux disease in patients with nontuberculous mycobacterial lung disease. Chest.

[11] Marchiori E, Zanetti G, Mano CM, Hochhegger B (2011). Exogenous lipoid pneumonia. Clinical and radiological manifestations. Respir Med.

[12] Hunter RL, Olsen M, Jagannath C, Actor JK (2006). Trehalose 6,6′-dimycolate and lipid in the pathogenesis of caseating granulomas of tuberculosis in mice. Am J Pathol.

[13] Hutchins GM, Boitnott JK (1978). Atypical mycobacterial infection complicating mineral oil pneumonia. JAMA.

[14] Couto SS, Artacho CA (2007). Mycobacterium fortuitum pneumonia in a cat and the role of lipid in the pathogenesis of atypical mycobacterial infections. Vet Pathol.

[15] Goren MB (1972). Mycobacterial lipids: selected topics. Bacteriol Rev.

[16] Burke DS, Ullian RB (1977). Megaesophagus and pneumonia associated with Mycobacterium Chelonei. A case report and a literature review. Am Rev Respir Dis.

[17] Cordier JF (2000). Organising pneumonia. Thorax.

[18] Keane MA, Hansell DM, Hind CR (1995). Wandering consolidation. Postgrad Med J.

[19] Miyagawa Y, Nagata N, Shigematsu N (1991). Clinicopathological study of migratory lung infiltrates. Thorax.

[20] Van Bleyenbergh P, Nemery B, Nolard N, Demedts M (2001). Recurrent flu-like illness with migrating pulmonary infiltrates of unknown aetiology. Respir Med.

[21] Ribo P, Pacheco A, Arrieta P, Teruel C, Cobeta I (2014). Gastroesophageal reflux as a cause of chronic cough, severe asthma, and migratory pulmonary infiltrates. Respirol Case Rep.

[22] Kunikane H, Shimizu T, Kusaka H, Abe S, Kuze A, Kawakami Y (1991). Pulmonary nontuberculous mycobacteriosis showing wandering shadows in chest roentgenograms. Respiration.

[23] Nakanaga K, Hoshino Y, Era Y, Matsumoto K, Kanazawa Y, Tomita A (2011). Multiple cases of cutaneous Mycobacterium Massiliense infection in a “hot spa” in Japan. J Clin Microbiol.

[24] Asakura T, Ishii M, Kikuchi T, Kameyama K, Namkoong H, Nakata N (2016). Disseminated Mycobacterium Marinum infection with a destructive nasal lesion mimicking Extranodal NK/T cell lymphoma: a case report. Medicine (Baltimore).

